# Combination treatment of T1-44, a PRMT5 inhibitor with Vactosertib, an inhibitor of TGF-β signaling, inhibits invasion and prolongs survival in a mouse model of pancreatic tumors

**DOI:** 10.1038/s41419-023-05630-5

**Published:** 2023-02-10

**Authors:** Eunji Hong, Wojciech Barczak, Sujin Park, Jin Sun Heo, Akira Ooshima, Shonagh Munro, Chang Pyo Hong, Jinah Park, Haein An, Joon Oh Park, Seok Hee Park, Nick B. La Thangue, Seong-Jin Kim

**Affiliations:** 1 GILO Institute, GILO Foundation, Seoul, Republic of Korea; 2 Department of Biological Sciences, Sungkyunkwan University, Suwon, Gyeonggi-do Republic of Korea; 3 Laboratory of Cancer Biology, Department of Oncology, University of Oxford, Old Road Campus Research Building, Old Road Campus, Oxford, UK; 4 Argonaut Therapeutics Ltd, Magdalen Centre, Oxford Science Park, Oxford, UK; 5 Theragen Bio Co., Ltd, Seongnam, Republic of Korea; 6 Department of Medicine, Samsung Medical Center, Sungkyunkwan University School of Medicine, Seoul, Republic of Korea; 7 Medpacto Inc., Seoul, Republic of Korea

**Keywords:** Targeted therapies, Epithelial-mesenchymal transition

## Abstract

Pancreatic ductal adenocarcinoma (PDAC) is the most lethal type of cancer and the third leading cause of cancer death with the lowest 5-year survival rate. Heterogeneity, difficulty in diagnosis, and rapid metastatic progression are the causes of high mortality in pancreatic cancer. Recent studies have shown that Protein arginine methyltransferase 5 (PRMT5) is overexpressed in pancreatic cancers, and these patients have a worse prognosis. Recently, PRMT5 as an anti-cancer target has gained considerable interest. In this study, we investigated whether inhibition of PRMT5 activity was synergistic with blockade of TGF-β1 signaling, which plays an important role in the construction of the desmoplastic matrix in pancreatic cancer and induces therapeutic vulnerability. Compared with T1-44, a selective inhibitor of PRMT5 activity, the combination of T1-44 with the TGF-β1 signaling inhibitor Vactosertib significantly reduced tumor size and surrounding tissue invasion and significantly improved long-term survival. RNA sequencing analysis of mouse tumors revealed that the combination of T1-44 and Vactosertib significantly altered the expression of genes involved in cancer progression, such as cell migration, extracellular matrix, and apoptotic processes. In particular, the expression of *Btg2*, known as a tumor suppressor factor in various cancers, was markedly induced by combination treatment. Ectopic overexpression of *Btg2* inhibited the EMT response, blocking cell migration, and promoted cancer cell death. These data demonstrate that the combination therapy of T1-44 with Vactosertib is synergistic for pancreatic cancer, suggesting that this novel combination therapy has value in the treatment strategy of patients with pancreatic cancer.

## Introduction

Pancreatic cancer is one of the most unconquered cancer types due to its difficulty in diagnosis and high mortality [1, 2]. The 5-year survival rate for pancreatic cancer is 11%, the lowest among all cancers. Pancreatic cancer also ranks as the third leading cause of death among cancer types with no improvement over the past two decades, despite significant advances in cancer treatment [3]. Since then, many therapies including combination chemotherapy have been tried to increase the survival rate of pancreatic cancer [4, 5]. For patients with pancreatic cancer, 5-fluorouracil-based adjuvant chemotherapy (ex. FOLFIRINOX) or gemcitabine-based combination therapy is commonly used and related clinical studies are also performed [6–8]. In addition, research on new treatments to overcome aggressive pancreatic cancer, such as epigenetic modification or immune system regulation, is being actively conducted [9–12].

PRMT5 is a major type II protein arginine methyltransferase that specifically produces symmetric dimethyl arginine (SDMA). PRMT5 is mostly upregulated and has multiple roles in cancer, including histone methylation, transcriptional regulation, signal transduction, DNA damage, and splicing [13–18]. PRMT5 as an anti-cancer target has gained significant interest in recent years and high levels of PRMT5 protein are associated with worse survival outcomes across multiple cancer types, including pancreatic ductal adenocarcinoma (PDAC) [19–24]. Particularly, inhibition of PRMT5 reduces the tumor growth via Myc pathway and inhibits epithelial-mesenchymal transition through EGFR-β-catenin axis in pancreatic cancer [19, 20]. In this study, we used T1-44, a selective small molecule inhibitor for PRMT5 protein. T1-44 is a similar derivative of EPZ015666, an inhibitor of PRMT5 enzyme activity that can be administered orally [21, 22]. Barczak et al. demonstrated the effect of T1-44 on cancer cell migration and invasion through regulation of the PRMT5-E2F axis and cell motility-related proteins [22].

In a previous study, it was known that histone H3 and H4 methylation by PRMT5 in combination with MEP50 regulates the transcription of genes involved in cancer cell metastasis and EMT response related to TGF-β signaling [23]. We therefore hypothesized that co-inhibiting PRMT5 activity and the TGF-β signaling pathway would be a powerful approach for PDAC treatment. The TGF-β signaling inhibitor, Vactosertib, is being actively studied as a combination therapy for several aggressive cancers [24, 25]. Our previous study demonstrated that the combination of Vactosertib + nal-irinotecan/5-fluorouracil/leucovorin was effective for cell migration and invasion in a preclinical model of PDAC [26]. Based on these preclinical studies, various clinical trials are currently underway in combination with Vactosertib for drug treatment for various cancers such as non-small cell lung cancer, advanced colorectal cancer, multiple myeloma, and pancreatic cancer [27–31].

Here, we proposed a robust combination therapy strategy for aggressive pancreatic cancer that increases the survival rate in preclinical mouse models. We found that the combination of Vactosertib and T1-44 blocked tumorigenesis and invasion, leading to increased survival.

## Results

### Effectiveness of PRMT5 inhibitor on pancreatic tumor growth

**Fig. 1:**
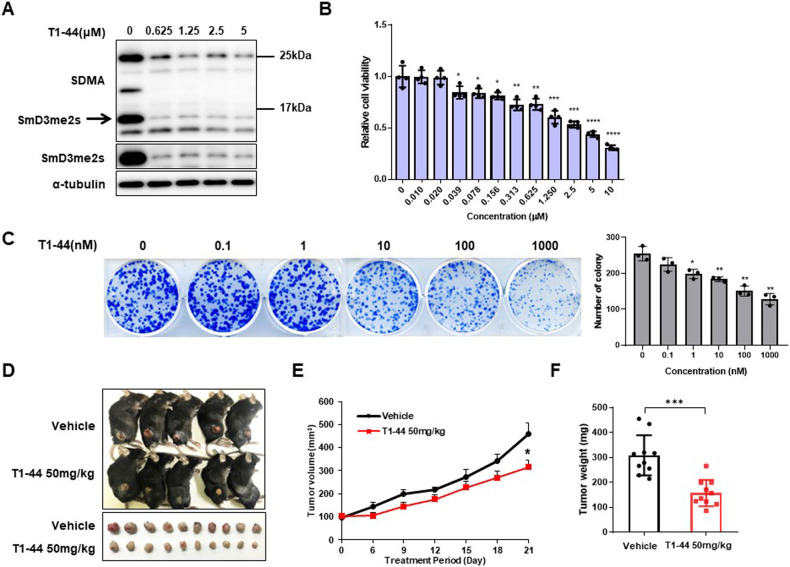
In vitro and in vivo response of T1-44. **A** Dose-dependent inhibition of symmetric arginine di-methylation of SmD3 by T1-44 in Panc02 murine pancreatic cancer cells. **B** Cell viability of Panc02 by T1-44 measured by MTT assay. Means with SD of OD values were presented relative to control. **C** Colony formation assay by treatment with various concentrations of T1-44. Colonies were counted after treatment of T1-44 for 2 weeks. **D** Administration of T1-44 (50 mg/kg, BID) to a subcutaneous tumor model of Panc02 cells. Individual tumor tissue was represented by tumor size for each group (bottom panel). **E** Graph of tumor volume measured every 3 days. **F** Tumor weights of vehicle and T1-44 treated groups at endpoint.

We first evaluated the effect of T1-44, a selective PRMT5 inhibitor, in pancreatic cancer. T1-44 inhibited symmetrical arginine dimethylation in both murine pancreatic cancer cells Panc02 and human pancreatic cancer cells SNU2491 (Fig. 1A, Supplementary Fig. S1A). To determine the ability of T1-44 in cell death and proliferation, an MTT assay was performed with a dose-dependent treatment for 6 days (Fig. 1B, Supplementary Fig. S1B). The cell viability of SNU2491 decreased to less than 50% in the low range of T1-44, and Panc02 also showed a response at more than 1 μM of T1-44. In addition, T1-44 inhibits the ability of cancer cells to form colonies (Fig. 1C, Supplementary Fig. S1C). To confirm the in vivo antitumor activity of T1-44, we subcutaneously injected Panc02 cells into C57BL/6 mice and administered T1-44 50 mg/kg twice a day (BID). T1-44 treatment slightly suppressed tumor growth rate and reduced final tumor volume and weight. (Fig. 1D, E, F). We also tested the same experiments using SNU2491 human pancreatic cancer cell line and immunodeficient mice NRGA (Supplementary Fig. S2A, B, C). In summary, targeting PRMT5 activity through T1-44 treatment results suppression of tumor growth in pancreatic tumor growth.

### Synergetic effect of TGF-β inhibitor and PRMT5 inhibitor combination in tumor progression

**Fig. 2:**
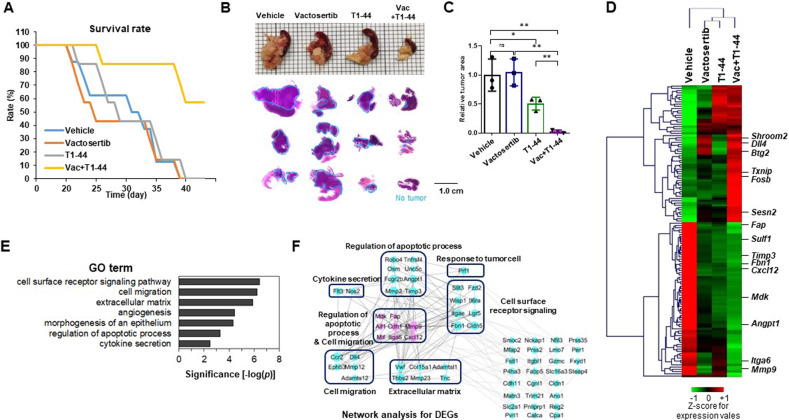
Vactosertib and T1-44 combined treatment for syngeneic orthotopic tumor model of C57BL/6-Panc02 and RNA-sequencing analysis of tumor tissues. **A** Survival rate plot of Panc02 syngeneic mouse model. Four groups (vehicle, Vactosertib, T1-44 mono-treatment and combination treatment) were calculated the percentage of live/total mice number for 47 days. **B** Representative picture of pancreas primary tumors with normal pancreas and spleen (top). Histological images (H&E) of tumor tissues distinguish between tumor and normal pancreas by blue lines (bottom). **C** Calculation of relative tumor area using H&E scan of four group. **D** Heatmap, **E** GO term analysis, and **F** Network analysis results of DEGs between Vactosertib+T1-44 and control from RNA sequencing analysis.

To synchronize the tumor microenvironment with pancreatic cancer patients, we established a syngeneic orthotopic model of Panc02 mouse pancreatic ductal adenocarcinoma cells and C57BL/6 mice, followed by oral administration 50 mg/kg of T1-44 (BID). Unexpectedly, T1-44 could not improve the survival rate of orthotopic tumor model (Supplementary Fig. S3A). We detected aggressive invasion of tumor cells and desmoplastic stromal tissue formation in pancreatic tumor lesions both in vehicle and T1-44 treated groups (Supplementary Fig. S3B). These phenomena are the major features of malignant pancreatic cancer and TGF- β signaling is one of the most crucial pathways for managing them [32, 33]. According to previous studies, Vactosertib, an effective TGF-β signaling inhibitor, incredibly inhibits invasion and desmoplastic stroma when combined with other anti-cancer drugs in pancreatic cancer [26, 34]. Therefore, we decided to treat T1-44 combined with Vactosertib on Panc02-C57BL/6 syngeneic orthotopic pancreas tumor model. When analyzed at the endpoint of the experiment, single administration of Vactosertib or T1-44 did not increase the survival rate of mice, whereas the combination treatment group of T1-44 and Vactosertib showed significantly improved results; 60% of mice survived more than 50 days (Fig. 2A). After 4 weeks of treatment, mice in each group were sacrificed and tumors localized to the pancreas and surrounding tissues were observed. There was no significant difference in the primary tumor size between the vehicle control group and the Vactosertib group, but the tumor size decreased in the T1-44 group alone. In particular, we found very small tumor tissue in the Vactosertib and T1-44 combination treatment group (Fig. 2B). We measured the relative tumor area in each group using section staining of tumor tissue. The average tumor area of the combined treatment group was reduced by more than 90% compared to the vehicle treatment group (Fig. 2C). Taking all the macroscopic results of the in vivo experiments, combination treatment of T1-44 with Vactosertib significantly reduced tumor growth and progression in a mouse pancreatic cancer model.

To determine which biological pathways were altered by the combination of Vactosertib and T1-44 and led to significant inhibition of cancer progression, we performed RNA sequencing analysis using primary tumor tissues from a mouse pancreatic cancer model. Differentially expressed genes (DEGs) between the co-treatment and vehicle groups were identified and visualized as heatmaps (Fig. 2D). Several cancer-associated genes were varied by Vactosertib and T1-44, in particular genes promoting cancer progression were highly downregulated and tumor suppressor upregulated compared to controls. Gene ontology (GO) terms with DEG were analyzed and rated by significance (Fig. 2E). GO terms related to cancer progression such as cell migration, extracellular matrix, angiogenesis, and apoptotic progression are placed at the top rank. Network analysis of DEGs revealed that the tumor suppressive effect of Vactosertib and T1-44 combination treatment alters gene expression primarily involved in the regulation of apoptotic processes and cell migration (Fig. 2F). The functional categories were highly interacted with cell surface receptor signaling, extracellular matrix, response to tumor cell, and cytokine secretion. In the network, eight genes related to the apoptotic process and cell migration, including *Mmp9*, *Cxcl12*, *Cdh1*, *Fap*, *Itga6*, *Mdk*, *Aif1*, and *Mif*, were downregulated with co-treatment, which may play vital roles in inhibition of cancer progression and cell death. Additionally, Prf1 exhibited remarkable up-regulation in Vactosertib and T1-44 co-treatment, probably suggesting the potential of natural killer cell activity [35]. Briefly, combination therapy of Vactosertib with T1-44 was effective in suppressing pancreatic cancer by regulating cancer cell migration and apoptosis processes.

### Inhibition of cancer cell motility and invasion with combination treatment in mouse pancreatic tumor model

**Fig. 3:**
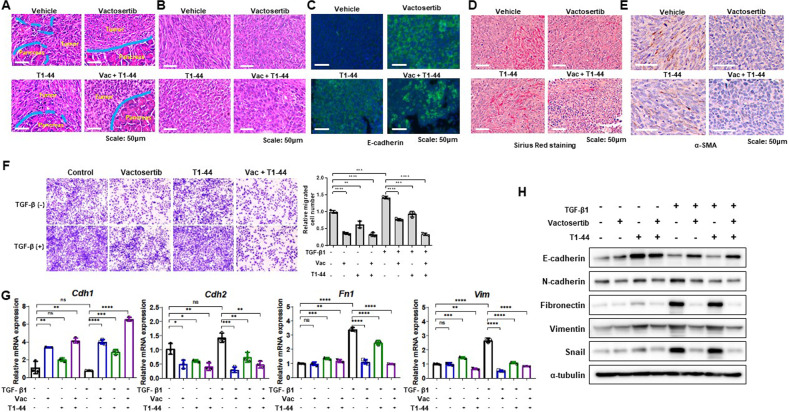
Inhibition of tumor invasion and fibrotic change of surrounding tissues through regulating EMT-related genes. **A** Histological analysis of pancreas tumor and normal tissues. Border lines between tumor and normal tissue were drawn by blue lines. Broken part of lines represents invading structure of tumor. **B** H&E images showing cell morphologies of each group. Spindle type (vehicle) and round type (Vactosertib, T1-44, and Vac+T1-44). **C** Immunofluorescence staining results of E-cadherin in tumor tissues. **D** Sirius Red staining indicating collagen deposition (strong red color) in tumor surrounding tissues. **E** α-SMA immunohistochemistry-stained tumor tissues (brown). **F** Panc02 cell migration assay using trans-well system. TGF-β1 (5 ng/ml), Vactosertib (Vac) (500 ng/ml), and T1-44 (5 μg/ml) were primarily treated for 48 h. **G** mRNA levels of EMT markers using quantitative PCR data. **H** Protein levels of EMT markers using western blotting.

We wonder why the survival rate of mice with orthotopic injection of Panc02 cells into the pancreas was significantly increased only in the Vactosertib and T1-44 combination treatment groups compared to the vehicle or single treatment groups. RNA sequencing data of pancreatic tumor tissue showed that the expression of genes related to cell migration or extracellular matrix was greatly altered by the combination treatment of Vactosertib and T1-44. Therefore, we histologically analyzed the pancreatic primary tumor and surrounding tissues to determine the effect of this combination on cancer cell invasion into surrounding tissues. When a primary tumor forms in the pancreas, cancer cells invade normal pancreatic tissue. Treatment with T1-44 reduced invasiveness to surrounding tissues, but some of the borders were destroyed by tumor cells. On the other hand, the Vactosertib alone or co-treated group showed a clear boundary between the tumor and normal tissue without an invaded morphology (Fig. 3A). Treatment with either Vactosertib or T1-44 also transformed the cancer cell morphology, mesenchymal (spindle) type, into an epithelial (round) cell type (Fig. 3B). Expression of E-cadherin, a representative marker of mesenchymal-epithelial transition (MET), explained the change by Vactosertib or T1-44 alone treatment in each group of tumor tissues, and suppressed EMT-induced cell invasion, and the combined treatment maximized the effect (Fig. 3C). Moreover, Sirius red staining and α-SMA immunohistochemical staining revealed tissue fibrosis enhancing cancer metastasis, and chemical resistance was significantly reduced in the Vactosertib and T1-44 combination treatment group (Fig. 3D, E). Pancreatic cancer cell migration in vitro was reduced by the combination treatment of Vactosertib and T1-44 and TGF-β-induced migration was also blocked by the combination treatment (Fig. 3F, Supplementary Fig. S4). Consistently, the mRNA and protein levels of TGF-β target genes, including EMT marker genes, regulated by TGF-β treatment, were significantly modulated when combination treatment compared with treatment with Vactosertib alone or T1-44 alone (Fig. 3G, H; Supplementary Fig. S5). Thus, these findings suggest that the combination treatment of Vactosertib with T1-44 significantly reduces cancer cell invasion and migration by inhibiting EMT both in vivo and in vitro.

### Regulation of apoptotic processes by Vactosertib and PRMT5 inhibitor co-treatment in the syngeneic mouse model of pancreatic cancer

**Fig. 4:**
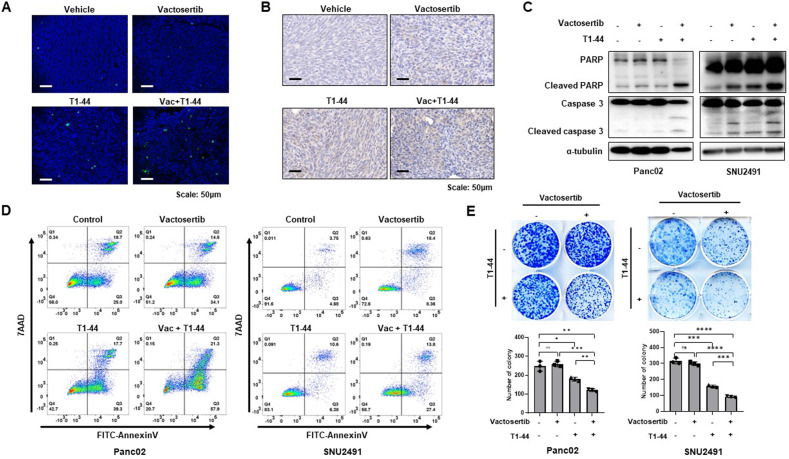
Positive regulation of apoptotic process by co-treatment of Vactosertib and T1-44. **A** TUNEL assay of primary tumor tissues from Panc02 mouse experiment. Green fluorescence dots indicate apoptotic positive cells. **B** Immunohistochemistry staining of cleaved-caspase3 **C**. Western blotting data of PARP and Caspase3. Cleaved forms of two protein indicate apoptosis signaling. **D** Flow cytometry of Annexin V-7AAD staining. **E** Colony forming assay of Panc02 and SNU2491 cell lines under treatment of Vactosertib and T1-44.

Another notable GO term for DEG in RNA sequencing is the regulation of apoptotic processes. Tumor tissue staining of TUNEL indicates that combination treatment of Vactosertib with T1-44 increased the number of positive apoptotic cells in mouse primary tissues of pancreatic cancer (Fig. 4A). In addition, cleaved form of Capase3 indicating signaling pathway of apoptosis was highest expressed in tumor of co-treated group (Fig. 4B). In vitro data from mouse and human pancreatic cancer cells also support that the combination treatment of Vactosertib and T1-44 positively regulated the apoptotic process, neither as a control nor as monotherapy. Cleaved PARP and cleaved Caspase3, representative markers of apoptosis, were significantly enhanced in the combination treatment group (Fig. 4C). Also, Annexin V staining results showed that the number of apoptotic cells was particularly abundant in the combination treatment group compared to the control group (Fig. 4D). In addition, cell colony formation and growth were decreased in the combination treatment group (Fig. 4E), suggesting that apoptosis plays a major role in effective pancreatic tumor reduction by combination therapy of Vactosertib and PRMT5.

### Elevated expression of tumor suppressor *Btg2* by combination treatment of Vactosertib and T1-44

**Fig. 5:**
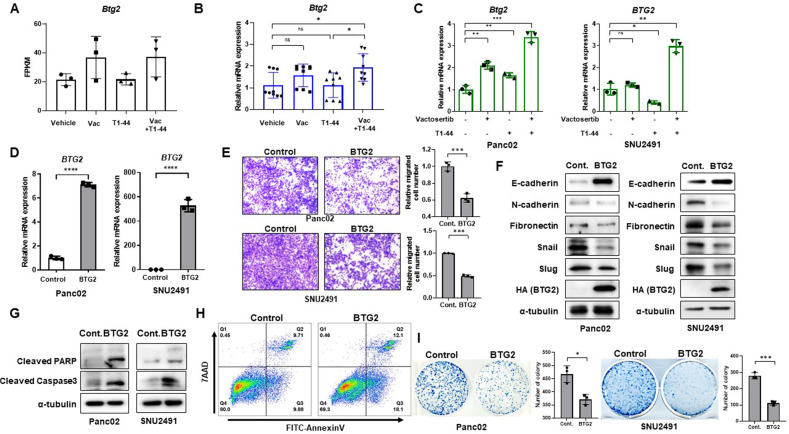
Upregulation of *Btg2* by Vactosertib and T1-44 combined treatment, suppressing cell migration and promoting apoptosis. **A** FPKM value of *Btg2* from RNA sequencing. **B** qPCR validation using mRNA from combination treated-tumor tissues and **C** co-treated Panc02 and SNU2491 cell lines. **D** Relative mRNA expression of *BTG2* **E** Cell migration assay, **F** Protein markers of EMT response, **G** apoptosis markers, **H** flow cytometry analysis of Annexin V, and colony formation assay with overexpressed stable cell lines.

Analysis of RNA sequencing data suggests several candidates for co-therapeutic target genes of Vactosertib and T1-44. Among the candidates, we were interested in several genes involved in tumor progression or suppression and validated those genes using RNA from pancreatic tumor tissue and cancer cells. Expressions of *Btg2* and *Dll4*, previously studied as tumor suppressors, were upregulated in the Vactosertib and T1-44 combination treatment group (Fig. 5A–C; Supplementary Fig. S6). On the other hand, we identified repressed genes such as *Mdk*, *Fap*, *Mmp9*, *Itga6*, and *Cxcl12*, known as mediators of tumor growth, migration, invasion, and fibrosis (Supplementary Fig. S7-S9). Similarly, mRNA levels of candidate genes were similarly altered in Panc02 mouse cells and SNU2491 human cells under combination treatment (Fig. 5C, Supplementary Fig. S9).

Among the candidate lists of target genes, we focused on the Btg2 gene, which has been studied as a tumor suppressor whose expression is suppressed in several types of cancer. In the pancreatic adenocarcinoma TCGA data, BTG2 expression is significantly suppressed in tumor tissues compared to normal tissues (Supplementary Fig. S10A). Further, BTG2 expression and disease-free survival rate of pancreatic cancer is positively correlated (Supplementary Fig. S10B). We generated cell lines stably overexpressing BTG2 in Panc02 and SNU2491 (Fig. 5D). Ectopic expression of BTG2 suppressed cell migration and EMT-related markers in both cell lines (Fig. 5E, F). Moreover, positive regulation of the apoptotic process, represented by enhanced cleaved PARP, cleaved Capase3 expression (Fig. 5G) and annexin V-stained cell number, was also seen in BTG2 overexpressed cells (Fig. 5H). Cell colony formation was also reduced by overexpression of BTG2 (Fig. 5I). Collectively, various genes involved in cancer progression were regulated by the combination treatment of Vactosertib and T1-44, and in particular, BTG2 is a key regulator of EMT-induced inhibition of cell migration and increased tumor cell death.

## Discussion

Genetic mutations are the most well-known hallmarks of pancreatic cancer, and epigenetic processes are also important for cancer development and progression [36, 37]. The type II methyltransferase PRMT5 has diverse effects on cancer biological functions, such as histone methylation and inhibition or promotion of tumor-associated signaling pathways. Histone arginine methylation by PRMT5 attenuates oncogenic signaling pathways, thereby regulating transcriptional gene expression [38–40]. The arginine residue of E2F1, a key molecule in cancer cell growth, is the target of methylation by PRMT5 [41]. In lymphoma cells, PRMT5 activates the WNT/β-catenin and AKT/GSK3β signaling pathways and regulates target gene expression [42]. In addition, the tumorigenicity and aerobic glycolysis of pancreatic cancer is increased by PRMT5, mediating epigenetic silencing of E3 ligase FBW7 with upregulation of c-Myc [43]. A selective PRMT5 inhibitor, EPZ015666, has antitumor effect via reducing proliferative role of cancer cells and tumor growth in mantle cell lymphoma (MCL) xenograft model [21]. In this study, we attempted to investigate whether the combination treatment of T1-44, an inhibitor of PRMT5 methyltransferase activity, and Vactosertib, a TGF-β signaling inhibitor, was more effective in inhibiting cancer growth and metastasis and increasing survival rate than T1-44 or Vactosertib alone using human and mouse pancreatic cancer cells. Our in vitro and in vivo experiments demonstrated that combination treatment of T1-44 with Vactosertib significantly reduced tumor growth and invasion and increased survival in a mouse pancreatic cancer model. EMT marker gene expression regulation and apoptosis induction were more effective when T1-44 and Vactosertib were treated together than T1-44 alone.

The heterogeneity and frequent alteration of components of the tumor microenvironment are considered important factors of pancreatic cancer malignancy and chemoresistance [44–46]. Cancer stromal tissue consists of the extracellular matrix (ECM), which creates a physical barrier and reduces sensitivity to drugs [47, 48]. In addition, cancer-associated fibroblasts (CAFs) in the tissues surrounding the PDAC tumor promote cancer progression, such as tumorigenesis and metastasis [49–51]. TGF-β signaling is important for the differentiation and metabolism of CAFs and mediates crosstalk between tumor cells and stromal cells [52–54]. The collagen deposition and expression of α-SMA were highly exited around the tumor tissues from Panc02 mouse model and lessened by Vactosertib and T1-44 combination treatment. Moreover, TGF-β signaling is the key factor involved cancer invasion and metastasis, regulating EMT response and tumor-related transcript factors [55–57].

A previous study has shown that PRMT5 is essential for expression of marker genes for metastasis and the EMT and is required for the epigenetic mechanism of the TGF-β response [23]. Interestingly, TGF-β treatment in A549 cells dramatically increased the relative abundance of PRMT5 protein and altered EMT protein markers with loss of E-cadherin and increased expression of Vimentin and Snail, suggesting the possibility that PRMT5 mediates the expression regulation of the EMT marker gene of TGF-β. This study showed that treatment of A549 lung adenocarcinoma cells with a specific PRMT5 inhibitor, GSK591, suppressed the expression of TGF-β1-induced EMT protein markers such as Snail and Vimentin, treatment of T1-44 alone did not suppress the expression of PRMT5. However, in our study the expression of these marker genes was suppressed only in the case of co-treatment of T1-44 with Vactosertib in pancreatic cancer cells. Whether these differences are due to cancer type, cell lines, or different PRMT5 inhibitors remains to be investigated in the future. Nevertheless, inhibition of the TGF-β signaling pathway in cancer therapy is a promising therapeutic target to prevent cancer invasion [58, 59].

BTG2, a member of the antiproliferative BTG/Tob family, is known as a tumor suppressor in various cancer types [60, 61]. Loss of BTG2 promotes tumor growth and reduces survival in patients with triple-negative breast cancer [62]. BTG2 can inhibit tumor growth and progression in breast cancer by suppressing the tumor microenvironments by regulating the mTORc2-AKT1-NFAT1-PHLPP2 signaling cascade [63]. In non-small cell lung cancer, BTG2 is a promising target for increasing radiation sensitivity with increased apoptosis and may be an early prognostic gene [64]. In transcriptomic analysis of mouse pancreatic tumor tissue, we identified BTG2 as a tumor suppressor gene whose expression was significantly induced by treatment with both T1-44 and Vactosertib in a mouse pancreatic tumor model. We discovered that the overexpression of BTG2 could suppress cell migration, colony formation, and EMT response in Panc02 and SNU2491 pancreatic cancer cells.

In conclusion, this study demonstrates that the combination treatment of T1-44, a PRMT5 inhibitor, and Vactosertib, a TGF-β signaling inhibitor, showed impressive synergistic results in improving survival, inhibiting cell invasion and EMT, and stimulating the apoptosis pathway in pancreatic cancer. This combination therapy could be one of the promising clinical therapies for patients with metastatic pancreatic cancer.

## Material and methods

### Cell culture and reagents

C57BL/6 mouse-derived pancreatic cancer cell line Panc02 was provided from Medpacto Inc. (Seoul, Korea). The human pancreatic adenocarcinoma cell lines, SNU2491 and PANC-1, were obtained from Korean cell line bank (Seoul, Korea). The cell lines were cultured in Dulbecco’s Modified Eagle’s Medium (DMEM) or RPMI 1640 contained with 10% FBS and 100 U/ml penicillin and 100 μg/ml streptomycin (WELGENE, Korea).

Selective PRMT5 inhibitor (T1-44) was provided by Argonaut Therapeutics Ltd, Oxford, UK. Vactosertib was obtained from Medpacto Inc. T1-44 and Vactosertib were solubilized in DMSO for in vitro experiments and in artificial gastric fluid (2 g/L NaCl, 3.2 g/L pepsin, 0.06 M HCl) for in vivo experiments. Antibodies against Symmetric Di-Methyl Arginine Motif (SDMA, Cat. No. 13222), Snail (Cat. No. 3879), PARP (Cat. No. 9532), and Caspase-3 (Cat. No. 9662) were obtained from Cell Signaling Technology (Boston, USA). Antibodies for E-cadherin (Cat. No. 610181), N-cadherin (Cat. 610921), and Fibronectin (Cat. No. 610077) were purchased from BD Biosciences. Anti-HA (sc-7392) and anti-Slug (sc-166476) were obtained from Santa Cruz Biotechnology (Texas, USA).

### Cell viability assay

A total of 2 × 10^3^ of cancer cells were seeded on 96well plates and treated T1-44 for 72 h. We used 0.5 mg/ml of MTT solution (Sigma, Cat. No. M5655) for colorimetric assay of cell viability. We dissolved the cells in 100 µl DMSO and measured the 540 nm OD values of each well. All data were analysed by the mean of triplicate with SD.

### Colony formation assay

A total of 500 of Panc02 and SNU2491 were seeded on 6well plates and treated T1-44 or Vactosertib for 2 weeks. We changed the medium with chemicals every 2–3 days. After 2 weeks, colonies were fixed and stained with 2% methylene blue in 50% ethanol.

### Trans-well system for migration and invasion assays

Cancer cells were pre-treated with Vactosertib and T1-44 on 60 mm dishes for 48 h. Then, 5 × 10^4^ of cells were moved to trans-wells (Flacon, Cat. No. 353097) for migration assay. After 48 h, migrated cells were fixed with 70% ethanol and stained with 0.05% crystal violet.

### Western blotting

Vactosertib or T1-44 treated cells were collected and washed by PBS twice. The cell pellets were lysed with RIPA buffer (50 mM Tris-HCL, 10 mM NaCl, 1% NP-40, 0.5 sodium deoxycholate, 0.1% sodium dodecyl sulfate, and a protease inhibitor cocktail) on ice for 30 min. Lysed proteins were separated by SDS-PAGE gel and transferred to PVDF membranes (Millipore, Cat. No. IPVH00010). The membranes were incubated with primary antibodies for overnight. The blots were detected with AI600 system (GE Healthcare Life Sciences, UK). The full blots of cropped western blot images were presented in supplementary figures.

### RNA isolation, cDNA synthesis and qRT-PCR

RNA from tumor tissues and cancer cells were isolated with easy-BLUE Total RNA extraction kit (Promega, Cat. No. 17061). For reverse transcription, we used M-MLV reverse transcriptase (Promega, CAT. NO. M1705) with 2 μg of mRNA. Synthesized cDNA was used for quantitative RT-PCR with QuantStudio5 Real-Time PCR instrument (Applied Biosystems, Massachusetts, USA) and TOPreal qPCR 2xPreMIX (Enzynomics, Cat. No. RT500M). All the expressions were normalized with *Gapdh*.

### Animal study

For subcutaneous syngeneic and xenograft model, 6-week-old male C57BL/6 (SLC, Japan) and NRGA (JA bio. Korea) mice were used. A total of 5 × 10^6^ of Panc02 and 2 × 10^7^ of SNU2491 were mixed with VitroGel® Hydrogel Matrix (The well bioscience, USA), injected to under skin of right flank of mice. After the tumor size were reached to 100mm^3^, we started to inject 50 mg/kg of T1-44 orally as twice in a day (BID). We followed up the size of tumors and body weight of mice every 3 days. For orthotopic model of pancreas cancer, 3 × 10^6^ Panc02 cells mixed with Matrigel (Corning, Cat. No. 35631) were injected into the tail of pancreas duct of C57BL/6. The mice were randomized to four groups and orally injected vehicle (gastric fluid), 25 mg/kg Vactosertib (5 days in a week), 50 mg/kg T1-44 (BID), and their combination. All animal experiments were performed following the guidelines and regulations of animal ethics and approved by Woojung Bio Animal facility (Suwon, Korea).

### RNA sequencing and data analysis

To perform RNA sequencing (RNA-Seq), cDNA libraries were prepared from 1 μg total RNA of each sample with three replicates using the TruSeq Stranded mRNA Sample Prep Kit (Illumina, Inc., San Diego, CA, USA), according to the manufacturer’s instructions. After qPCR validation, libraries were subjected to paired-end sequencing with a 100 bp read length using an Illumina HiSeq 4000 platform, yielding an average of 37.1 million reads per library.

The quality of raw reads was assessed with FastQC (version 0.11.9); the quality scores were greater than Q30. Clean reads, that quality scores were ≥Q30, were processed were aligned to the mouse reference genome GRCm38.p4 mm10 using TopHat2 [65] (version 2.1.1) with a set of gene model annotation. Gene expression quantification was performed using Cufflinks [66] (version 2.2.1), and fragments per kilobase of transcript per million reads mapped (FPKM) was calculated as the expression value. Differential expression (DE) in other treatments, including Vactosertib, T1-44, and Vac+T1-44, compared to vehicle was analyzed by using DEGseq [67] (version 1.50.0) with a cutoff set at *P* < 0.01 and ≥ |1.5 | -fold change. Expression pattern of differentially expressed genes (DEGs) selected was visualized as heatmap by using MeV (http://mev.tm4.org), and expressions of genes were shown as Z-score for FPKM. Gene ontology (GO) enrichment analysis for DEG datasets was performed by DAVID [68] with a cutoff of *P* < 0.001. Interaction for genes categorized to the enriched GO terms was searched using STRING database [69] (https://string-db.org/) with default confidence score (≥0.4) and further analyzed using Cytoscape (www.cytoscape.org) on the basis of the degree of connectivity of the nodes.

### H&E staining and immunohistochemistry of tumor tissues

Primary and surrounding tissues of pancreatic tumor were fixed in 10% formalin and embedded in paraffin. Tissue slides were stained with haematoxylin and eosin and Sirius red (collagen deposition). For immunohistochemistry staining, anti-α-SMA (Novus, Cat. No. NBP1-30894) and cleaved-Caspase3 (Cell Signaling Tech. Cat. No. 9661) were used.

### TUNEL assay

To detect the apoptotic tumor cells in tumor tissues, DeadEnd™ Fluorometric TUNEL System (Promega, Cat. No. G3250) was used with manufacturer’s protocol. Briefly, the tumor tissue slides were deparaffinized and rehydrated with ethanol. The tissues were permeabilized and covered with equilibration buffer and rTdT buffer. SSC buffer was used for stopping the reaction and nuclei were stained with VECTASHIELD® Antifade Mounting Medium with DAPI (Vector lab, Cat. No. H-1200).

### Annexin V staining assay

Flow cytometry for annexin V staining was performed using EzWay Annexin V-FITC Apoptosis Detection Kit (KOMA Biotech, Cat. No. K29100). A total of 3 × 105 cells were stained with Annexin V-FITC and 7AAD following the protocol. Labelled cells were detected by BD FACS Canto II and analysed data using Flowjo 10.8 software (BD Biosciences).

### TCGA data

The box plot expression and disease-free survival rate analysis in TCGA data were obtained from PAAD (pancreatic adenocarcinoma) dataset in GEPIA2 database [70].

### Statistics

All the data were analyzed statistically using two-tailed and unpaired Student’s *t*-test. Data were presented as the mean with SD by GraphPad Prism 8 software. Significance of data was determined by *P*-values, which were labelled by * <0.05, ** <0.01, *** <0.001, and **** ≤0.0001.

## Supplementary information

Supplementary material

Reproducibility checklist

## Data Availability

The raw data for RNA sequencing generated in this study have been deposited in the NCBI SRA database under accession code SRR22996981—SRR22996992 (https://trace.ncbi.nlm.nih.gov/Traces/sra/sra.cgi?view=run_browser&run=SRR_number [i.e., SRR number: SRR22996981]).
