# Supplementary material for: Combination treatment of T1-44, a PRMT5 inhibitor with Vactosertib, an inhibitor of TGF-β signaling, inhibits invasion and prolongs survival in a mouse model of pancreatic tumors
Source: Cell Death Dis. 2023 Feb 10;14(2):93. doi: 10.1038/s41419-023-05630-5 (PMC9918730; doi:10.1038/s41419-023-05630-5)
Supplement: Supplementary file 1 — Supplementary material [file 41419_2023_5630_MOESM1_ESM.docx]

**Combination treatment of T1-44, a PRMT5 inhibitor with Vactosertib, an inhibitor of TGF-****β signaling, inhibits invasion and prolongs survival in a mouse model of pancreatic tumors**

Eunji Hong^1,2^, Wojciech Barczak^3^, Sujin Park^1^, Jin Sun Heo^1^, Akira Ooshima^1^, Shonagh Munro^4^, Chang Pyo Hong^5^, Jinah Park^1^, Haein An^1,2^, Joon Oh Park^6^, Seok Hee Park^2^, Nick B. La Thangue^3^, and Seong-Jin Kim^1,7*^

^1^GILO Institute, GILO Foundation, Seoul, 06668, Republic of Korea, ^2^Department of Biomedical Science, College of Life Science, Sungkyunkwan University, Suwon, Gyeonggi-do 16419 Republic of Korea, ^3^Laboratory of Cancer Biology, Department of Oncology, University of Oxford, Old Road Campus Research Building, Old Road Campus, Roosevelt Drive, Oxford, OX3 7DQ, United Kingdom, ^4^Argonaut Therapeutics Ltd, Magdalen Centre, Oxford Science Park, Oxford OX4 4GA, United Kingdom, ^5^Theragen Bio Co., Ltd, Seongnam, 13488, Korea, ^6^ Department of Medicine, Samsung Medical Center, Sungkyunkwan University School of Medicine, Seoul, Republic of Korea, and ^7^Medpacto Inc., Seoul 06668, Republic of Korea


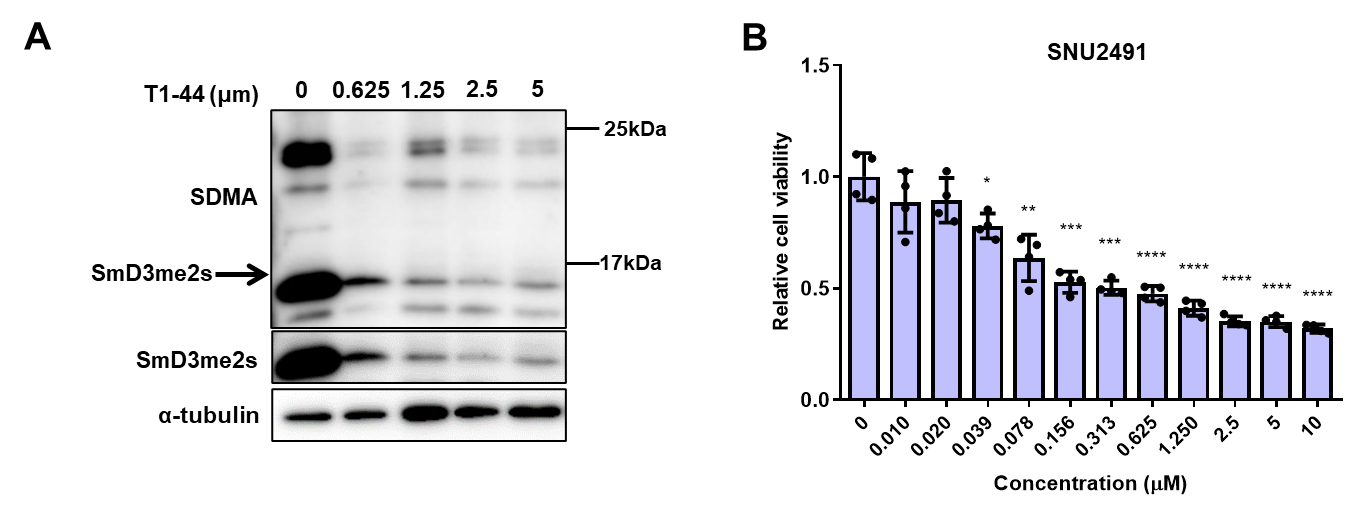


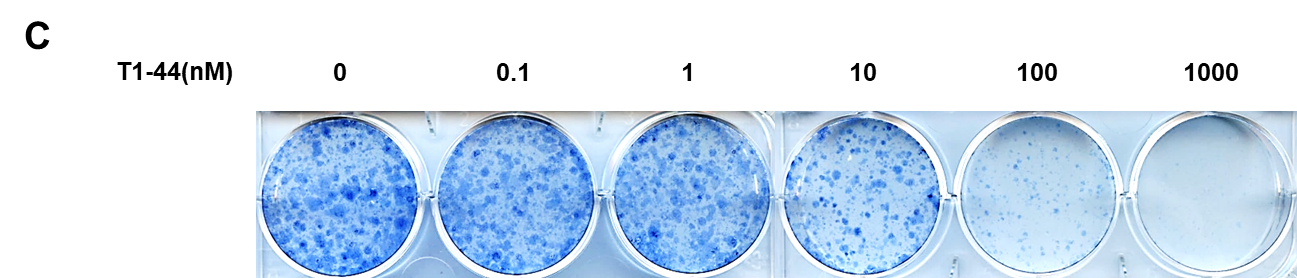

**Supplementary Fig. S1. Reduction of SDMA di-methylation and cell proliferation by T1-44 in SNU2491 cell**

**A**. Symmetric arginine di-methylation of SmD3 under treatment of T1-44. **B**. MTT assay for cell viability of SNU2491 with T1-44 treatment. Relative cell viability was presented by the mean with SD. **C**. Colony formation assay. The number of colonies were counted.


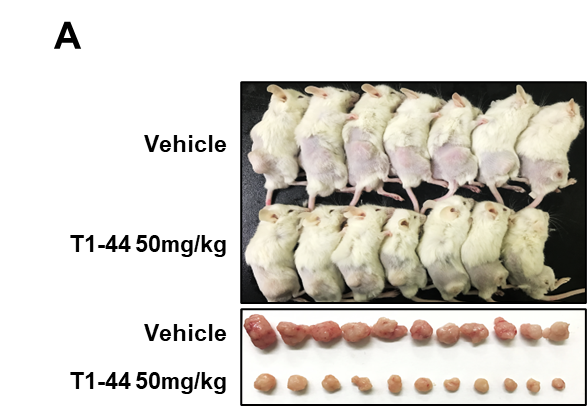


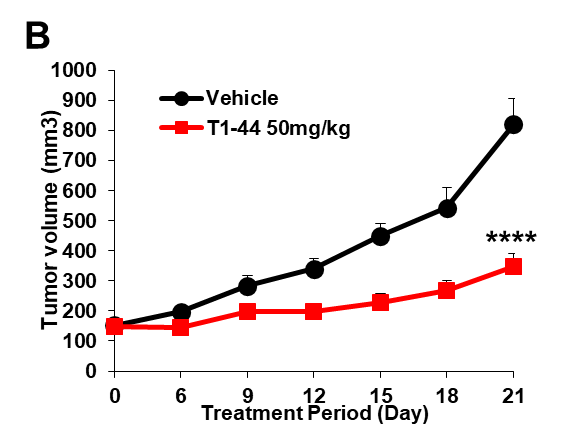

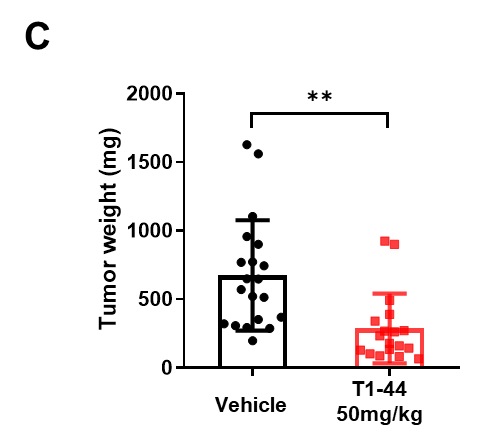


**Supplementary Fig. S2. *In vivo* tumor growth inhibition by T1-44 treatment in SNU2491 xenograft model**

**A.** Subcutaneous tumor model of SNU2491 were treated with T1-44 (50mg/kg, BID). Tumor tissues of vehicle and T1-44 were presented (bottom). **B**. The tumor volume measured every 3 days. P-value at the endpoint was marked on the graph. **C**. Tumor weight of two groups were presented by mean with SD.


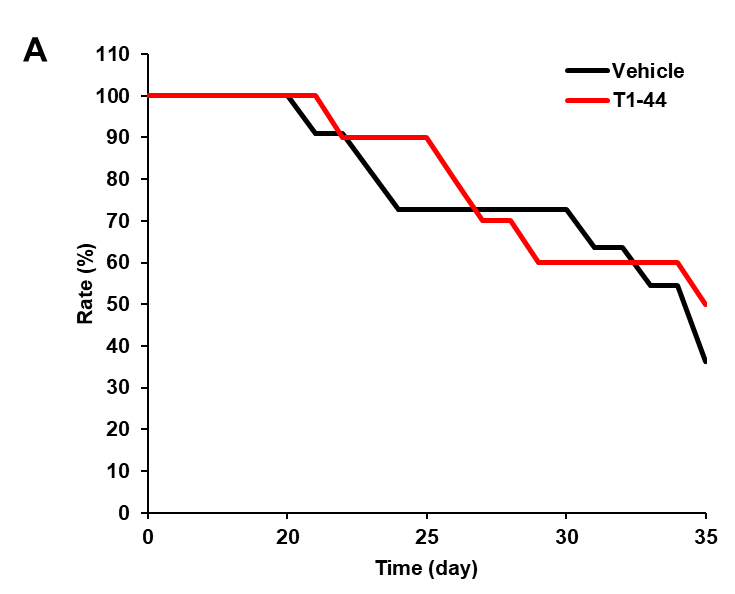


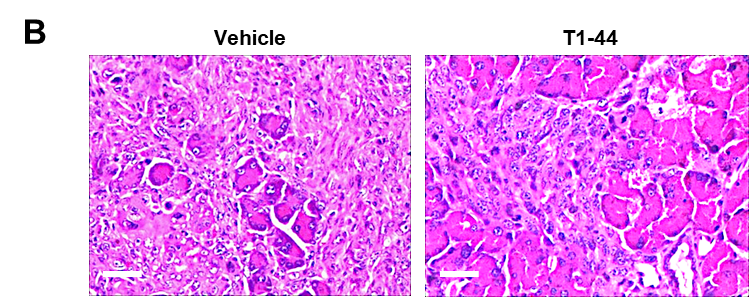


**Supplementary Fig. S3. T1-44 monotherapy for a syngeneic orthotopic mouse model of pancreatic cancer using panc02 cells**

**A**. The survival rate of panc02-C57BL/6 mouse syngeneic orthotopic model of pancreatic cancer, administered with T1-44 and vehicle. **B**. H&E staining of invaded pancreatic tumor and surrounding tissues.


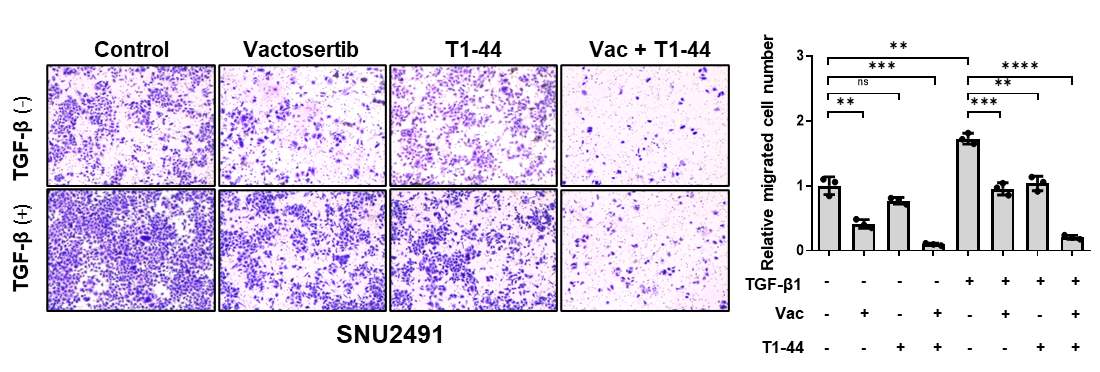


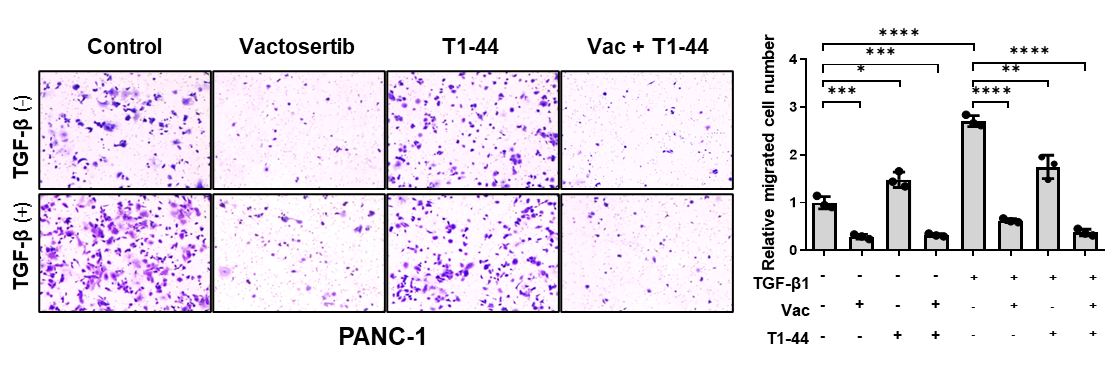


**Supplementary Fig. S4. TGF-β-induced cell migration was inhibited by Vactosertib and T1-44 co-treatment in human pancreatic cancer cells**

Cell migration experiment of Vactosertib (Vac) and T1-44 co-treatment under TGF-β1-treated condition using SNU2491 and PANC-1 cells. The number of migrated cancer cells were counted and displayed as relative values compared to control.


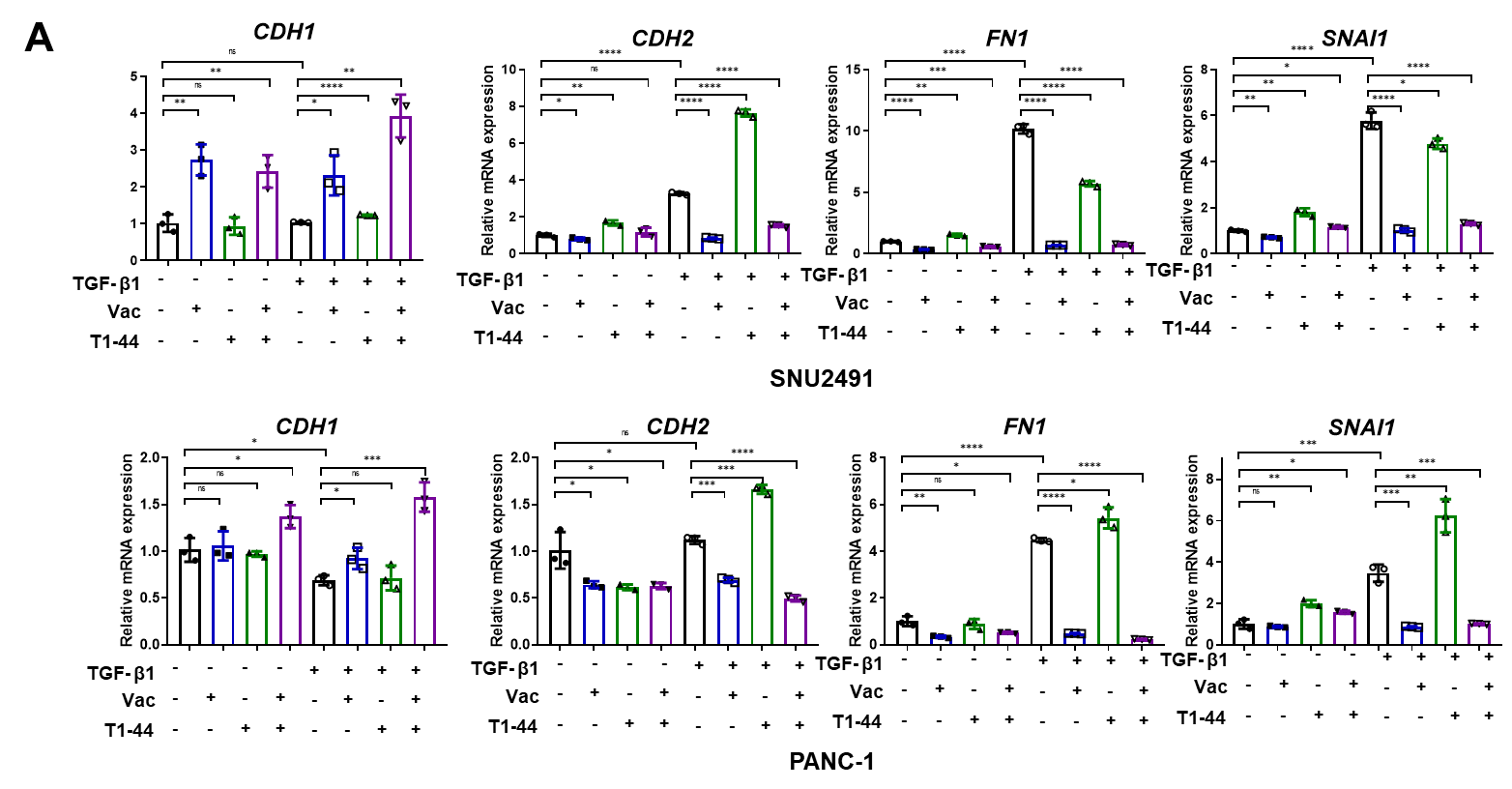


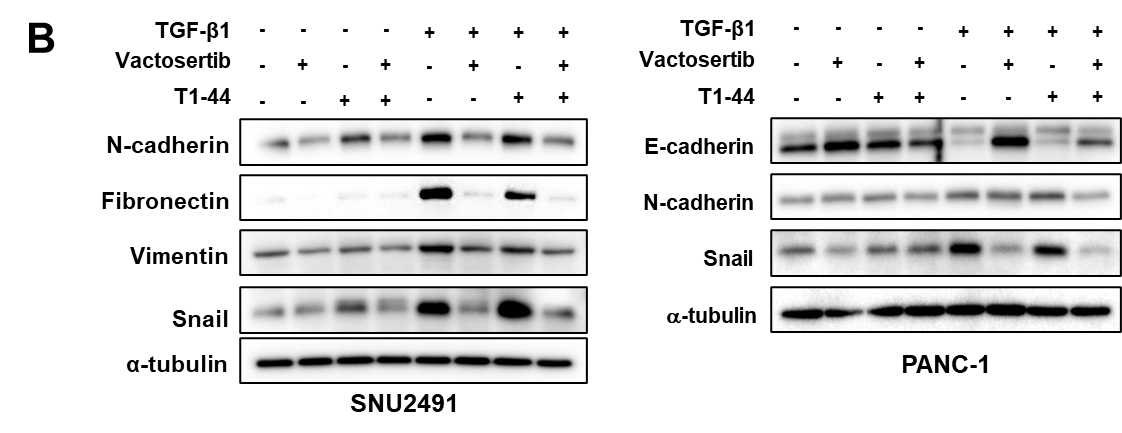


**Supplementary Fig. S5.** **Expression of TGF-β target genes, including EMT-related genes, regulated by TGF-β treatment were downregulated by combination treatment of T1-44 with Vactosertib.**

In human pancreatic cancer cell lines, SNU2491 and PANC-1, **A**. mRNA expression levels and **B**. protein levels of EMT-related genes were estimated by qRT-PCR and western blotting.


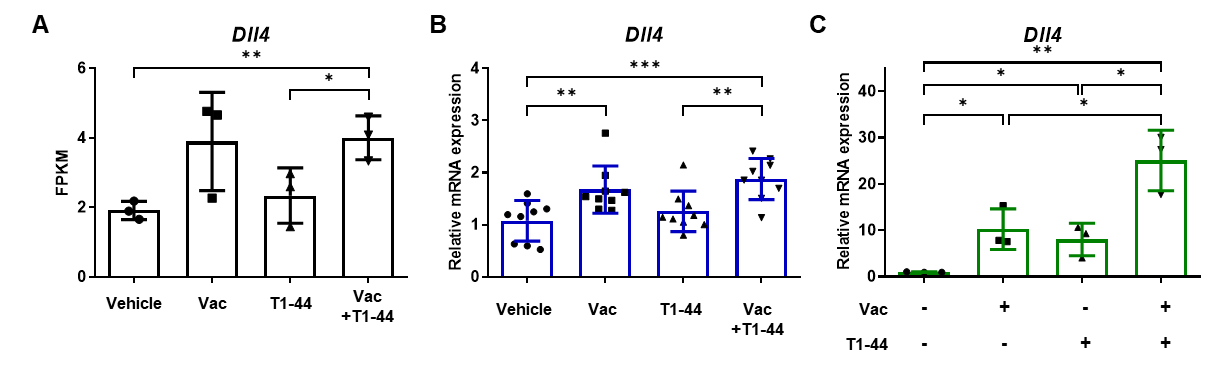


**Supplementary Fig. S6. FPKM value from RNA-seq and relative mRNA expression of tumor suppressor gene, *Dll4*, in tumor tissues and mRNA expression of *Dll4* gene in panc02 cells.**

**A**. FPKM values of *Dll4* gene from DEG analysis using tumor tissue RNA-seq data. **B**. Relative mRNA expression of *Dll4* gene in tumor tissues. **C.** Relative mRNA expression of *Dll4* gene in Panc02 cells treated with Vactosertib alone, T1-44 alone, or both Vactosertib and T1-44 by qRT-PCR. All data were presented as mean with SD.


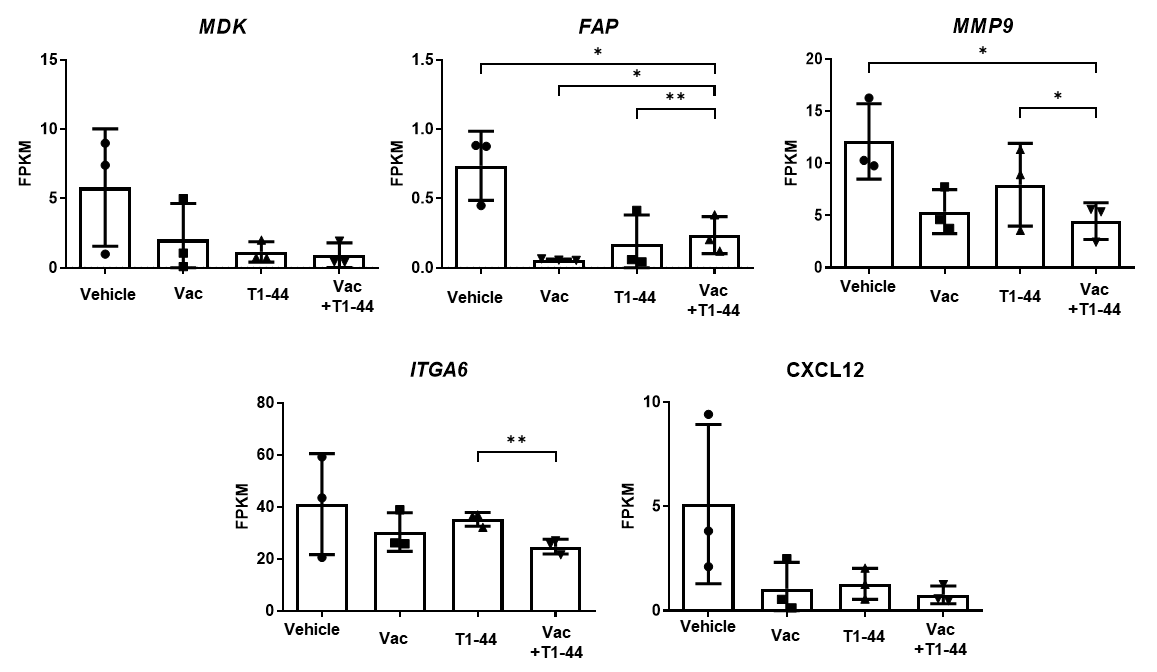


**Supplementary Fig. S7. FPKM values of genes** **known as mediators of tumor growth, migration, invasion, and fibrosis from RNA-seq in tumor tissues**

FPKM values of genes known as mediators of tumor growth, migration, invasion, and fibrosis from DEG analysis using tumor tissue RNA-seq data. All data were presented as mean with SD.


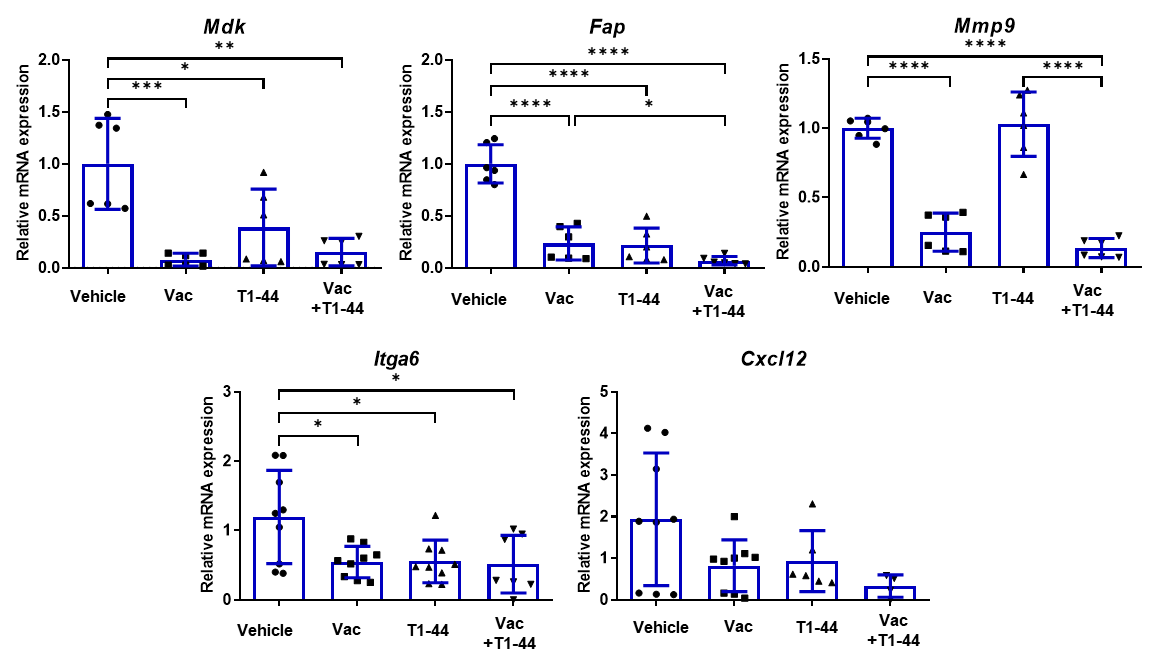


**Supplementary Fig. S8. mRNA expression of genes known as mediators of tumor growth, migration, invasion, and fibrosis in tumor tissues**

Relative mRNA expression of genes known as mediators of tumor growth, migration, invasion, and fibrosis in tumor tissues by qRT-PCR. All data were presented as mean with SD.


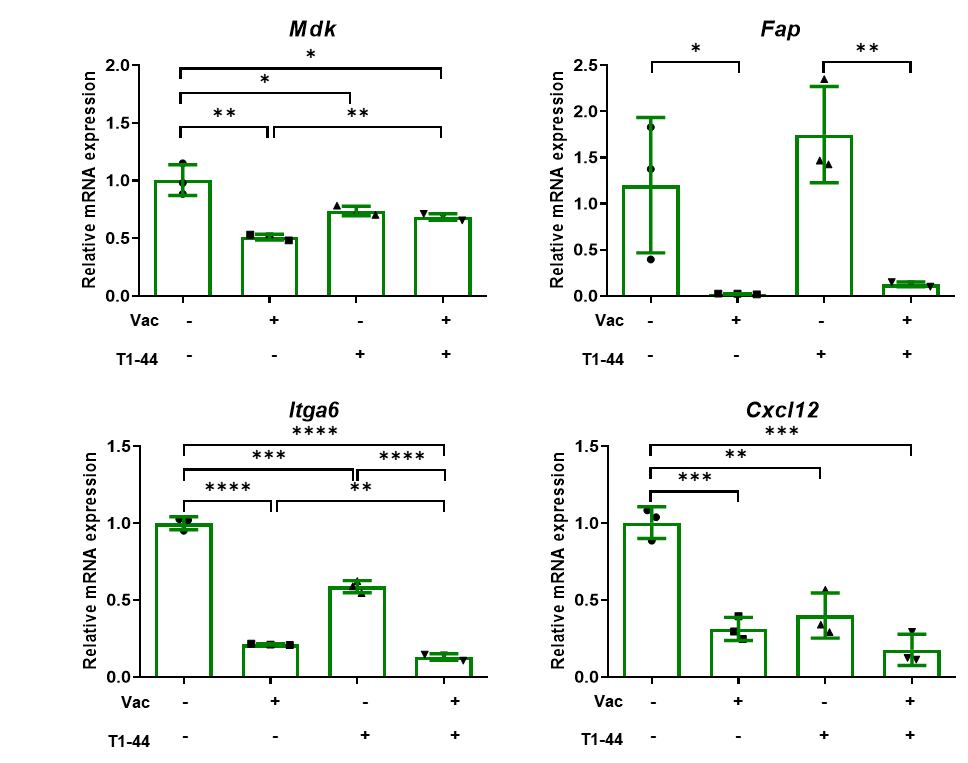


**Supplementary Fig. S9. mRNA expression of genes known as mediators of tumor growth, migration, invasion, and fibrosis from RNA-seq in Panc02 cell**

Relative mRNA expression of genes known as mediators of tumor growth, migration, invasion, and fibrosis in Panc02 cells treated with Vactosertib alone, T1-44 alone, or both Vactosertib and T1-44 by qRT-PCR. All data were presented as mean with SD.


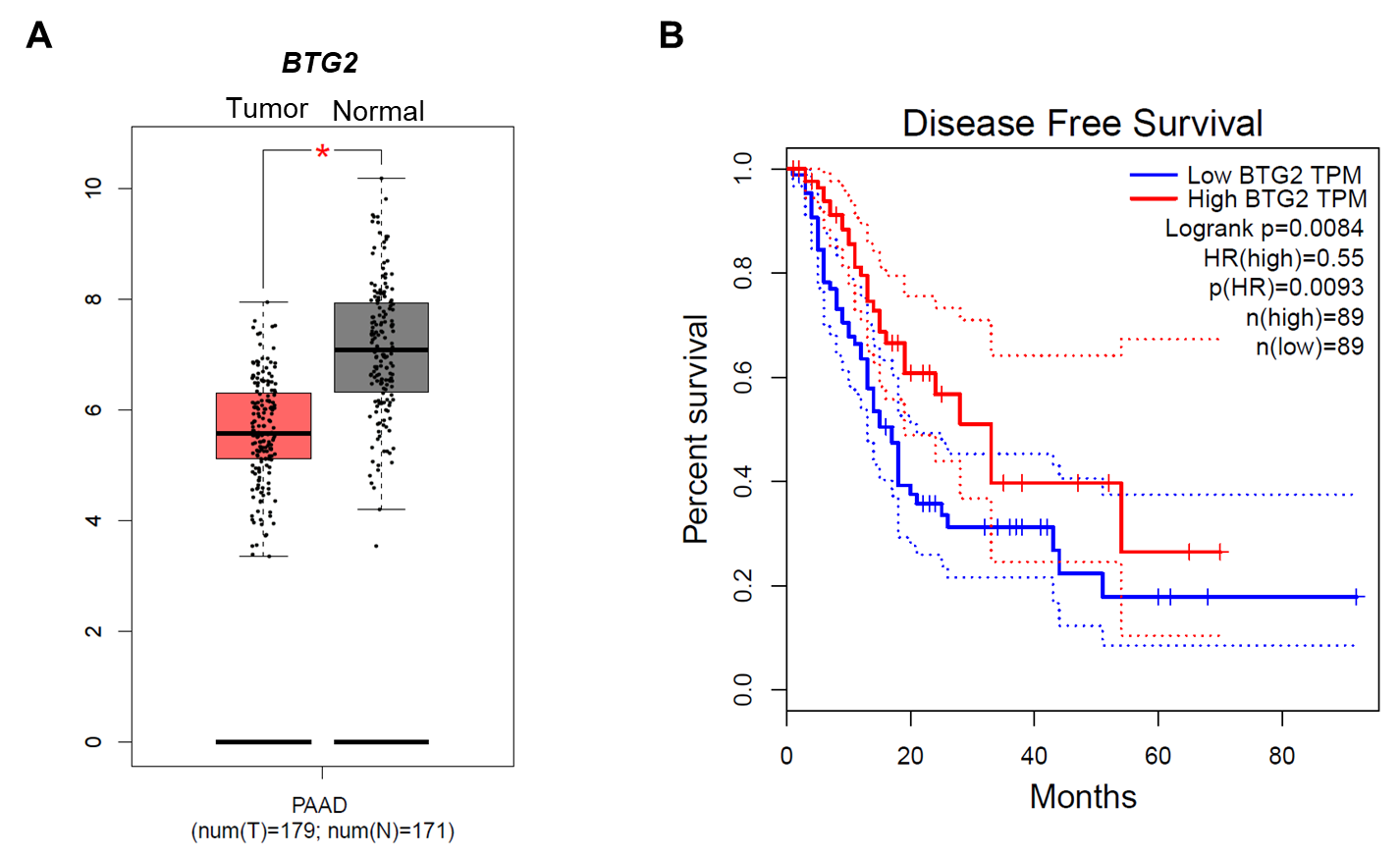


**Supplementary Fig. S10. Suppressed *BTG2* expression and correlation with disease free survival in pancreatic cancer TCGA data**

**A**. Boxplot of *BTG2* expression comparison between normal and tumor tissues in pancreatic adenocarcinoma. The number of tumor samples is 179 and normal is 171. **B**. Correlation with BTG2 expression and disease-free survival rate of pancreatic adenocarcinoma.


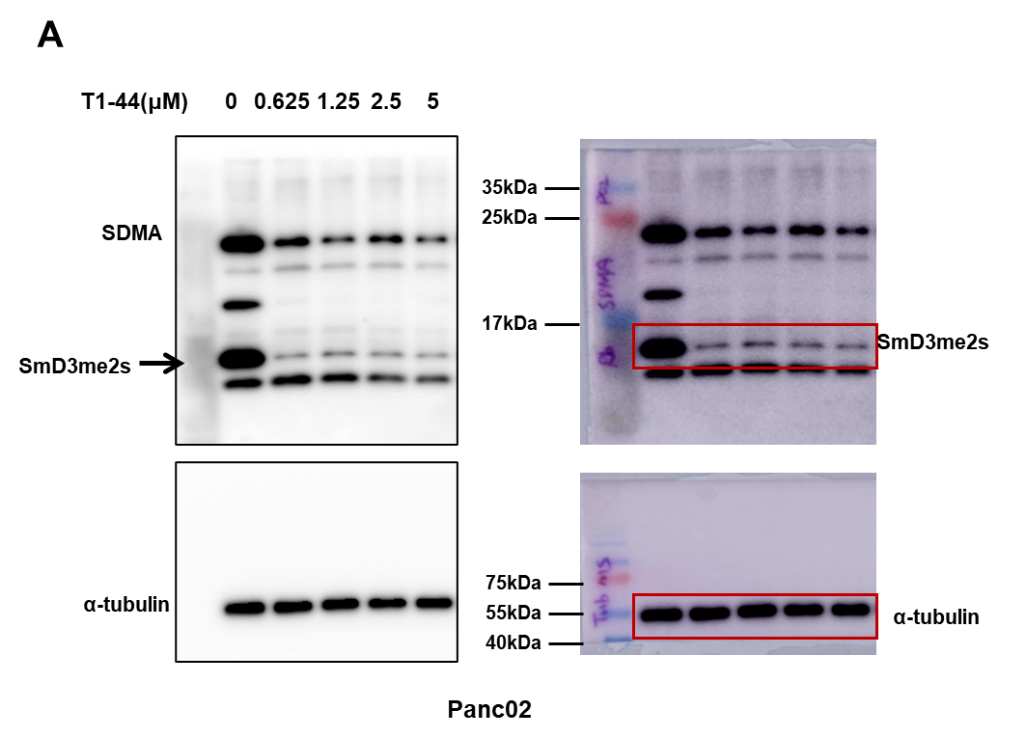


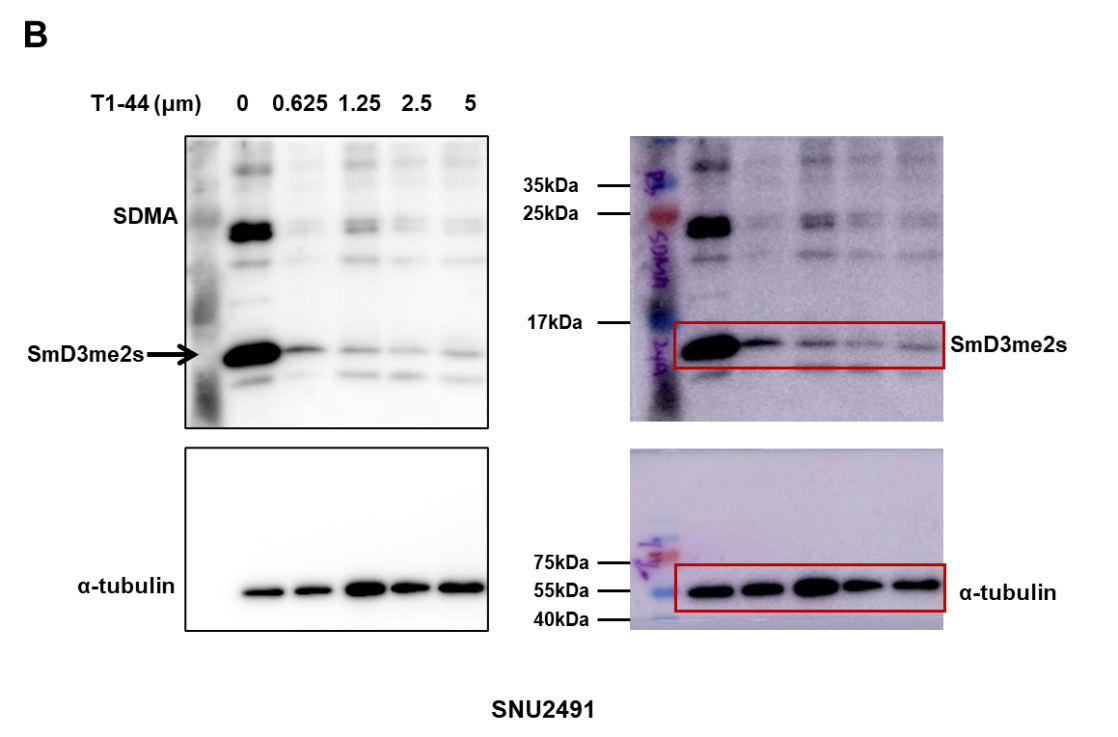


**Supplementary Fig. S11. Uncropped western blots for Fig. 1A and Supplementary Fig. S1A**


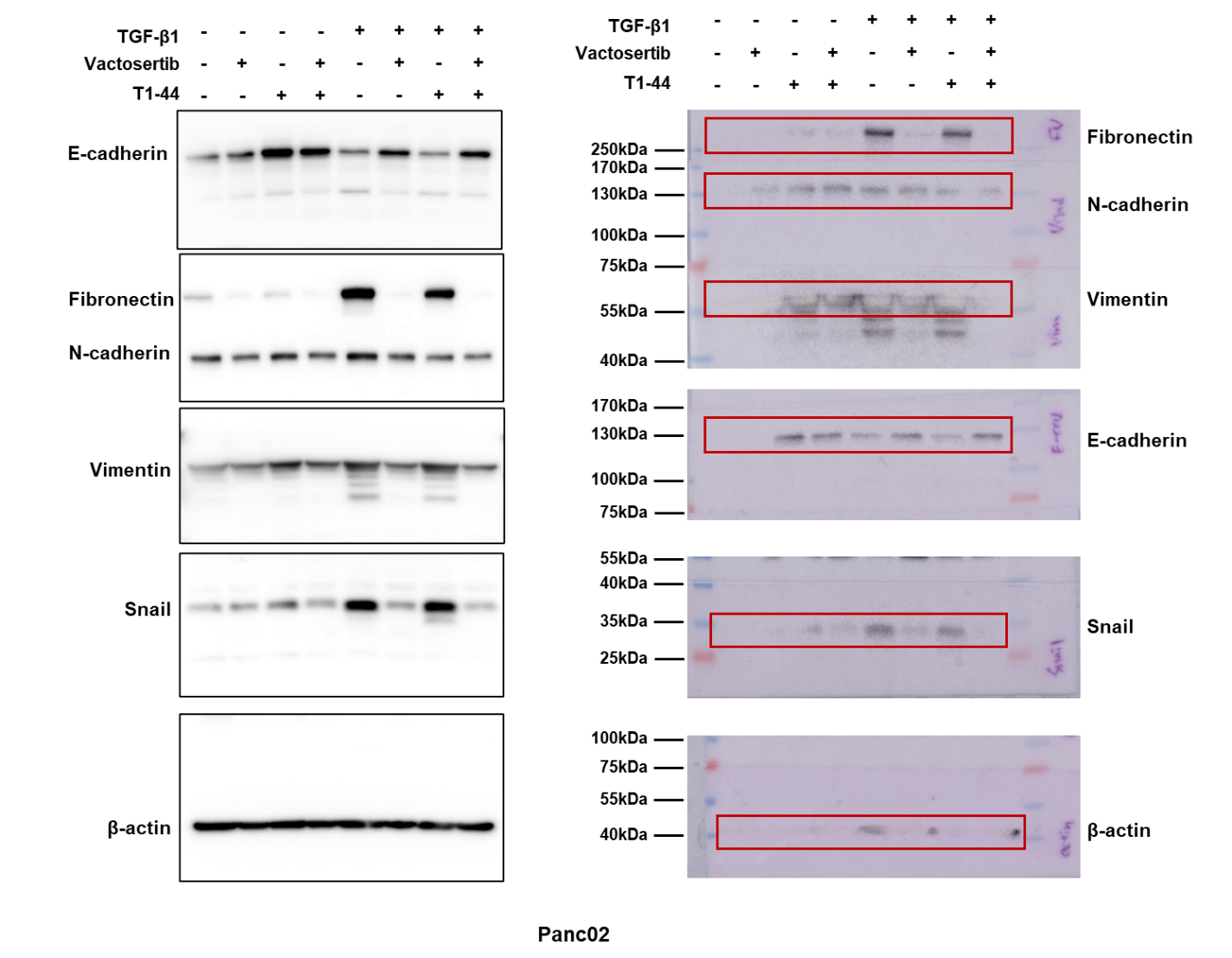


**Supplementary Fig. S12. Uncropped western blots for Fig. 3H**


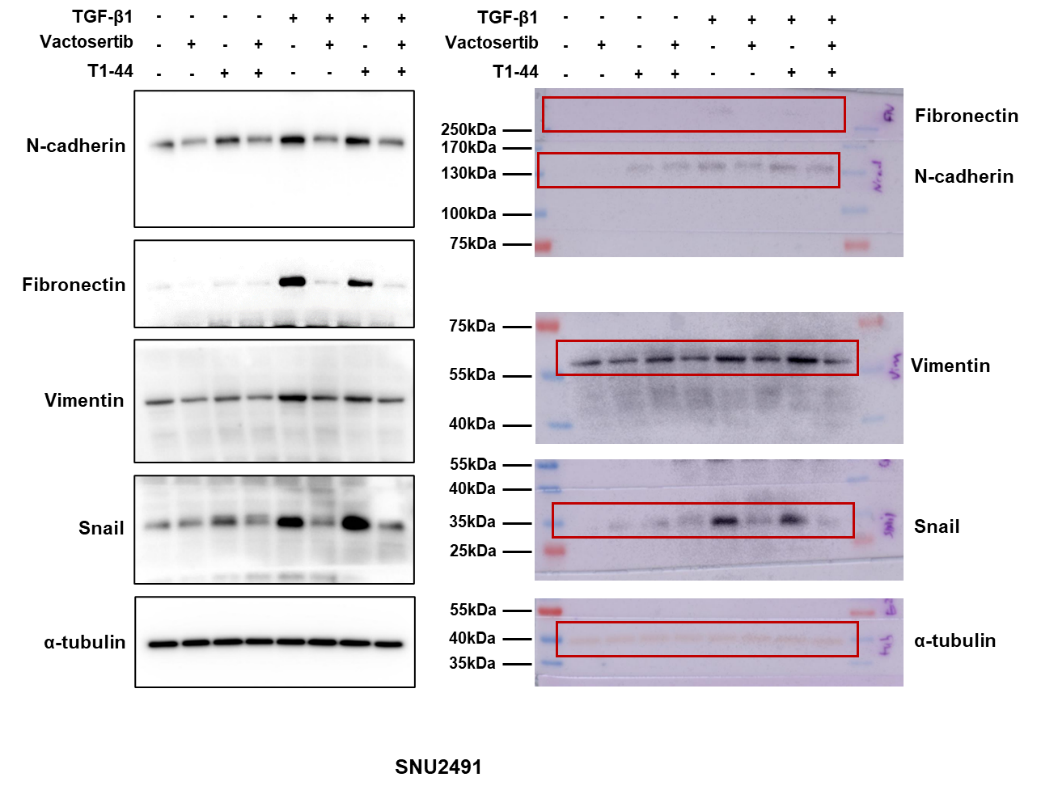


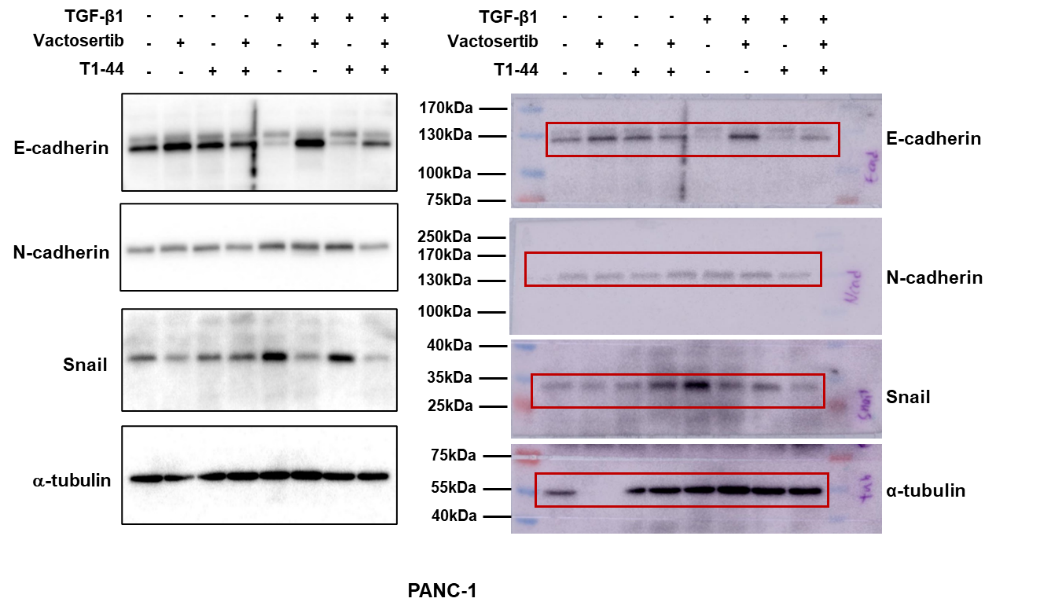


**Supplementary Fig. S13. Uncropped western blots for Supplementary Fig. S5B**


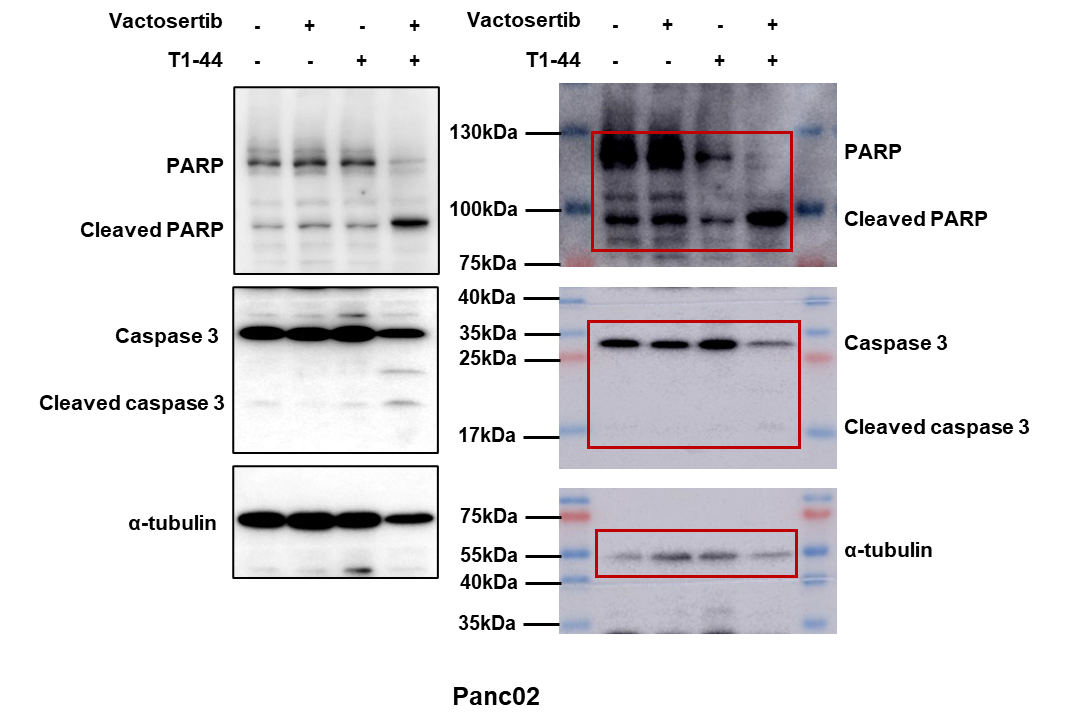


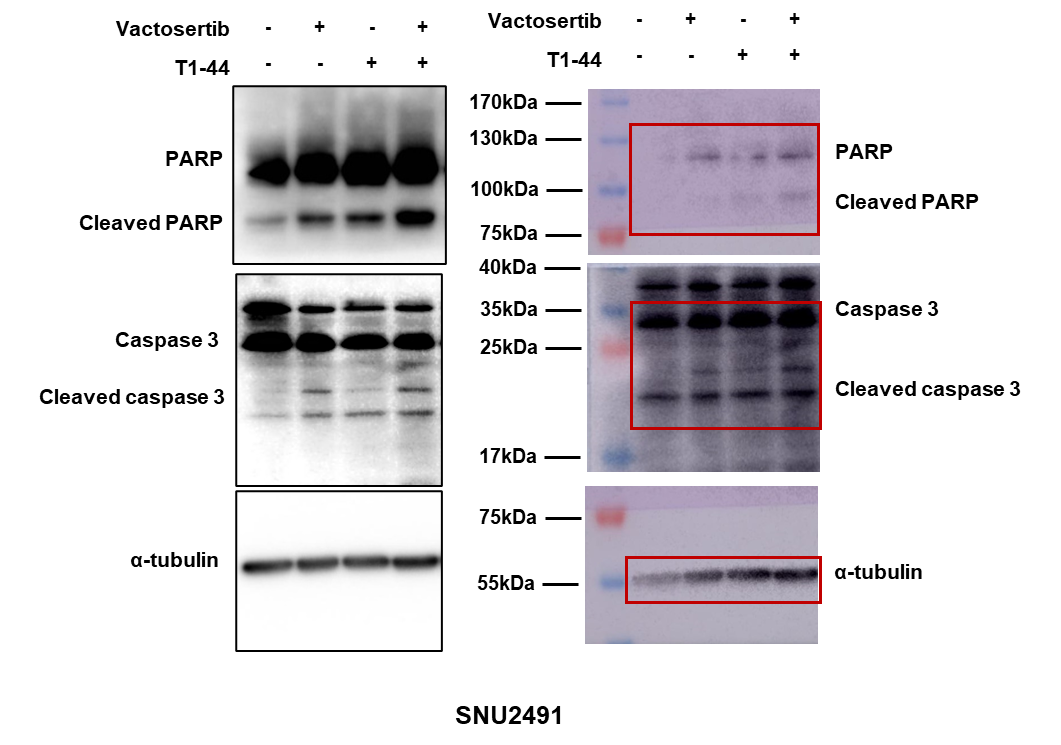


**Supplementary Fig. S14. Uncropped western blots for Fig. 4C**


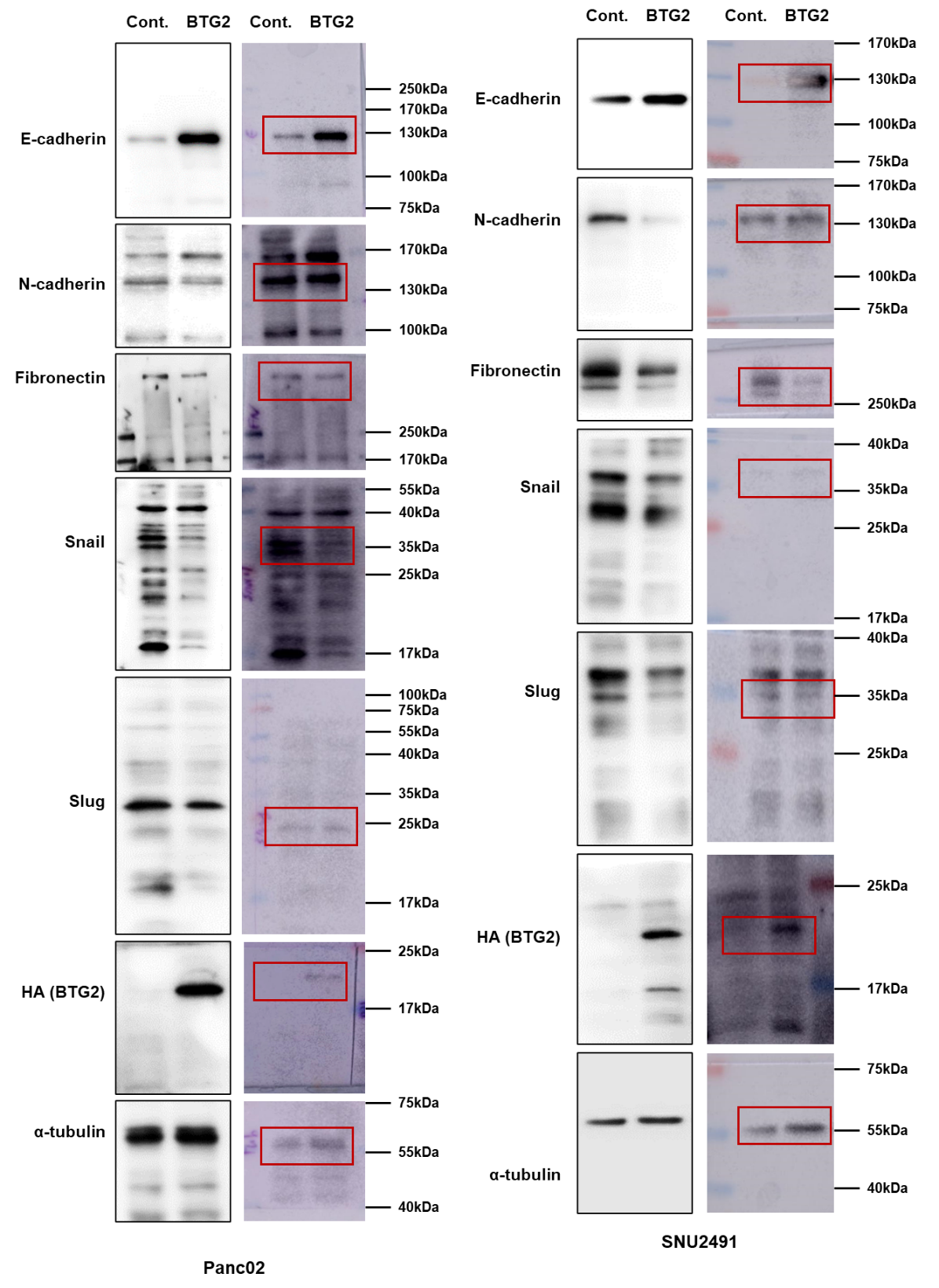


**Supplementary Fig. S15. Uncropped western blots for Fig. 5F**


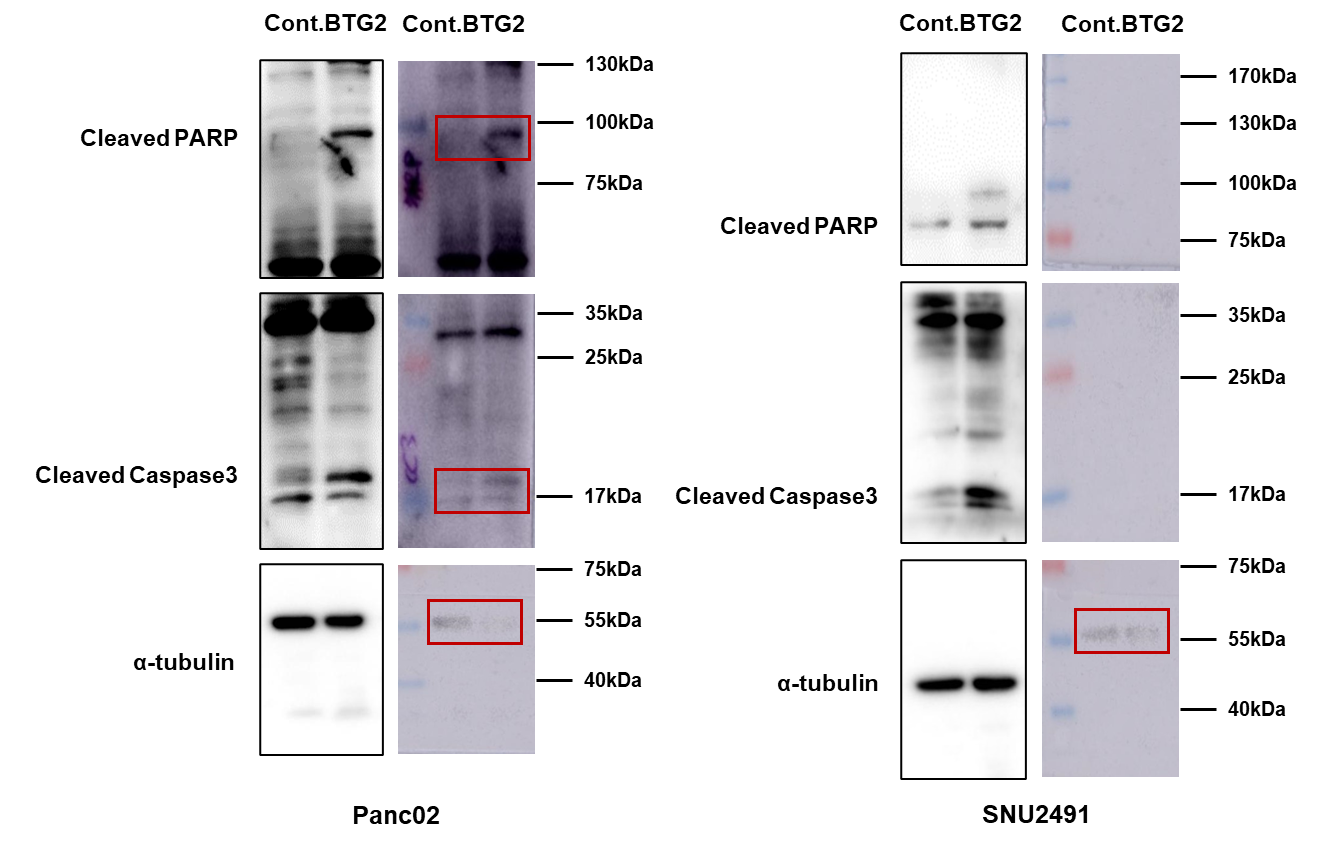


**Supplementary Fig. S16. Uncropped western blots for Fig. 5G**
